# Survival and prognostic differences between appendiceal NETs and goblet cell adenocarcinomas

**DOI:** 10.1530/EO-25-0028

**Published:** 2025-08-08

**Authors:** Kim Dijke, José van den Berg, Koert F D Kuhlmann, Sonja Levy, Gerlof D Valk, Margot E T Tesselaar

**Affiliations:** ^1^ Department of Gastrointestinal Oncology, Netherlands Cancer Institute, Amsterdam, The Netherlands; ^2^ Department of Endocrine Oncology, University Medical Centre Utrecht, Utrecht, The Netherlands; ^3^ Department of Pathology, Netherlands Cancer Institute, Amsterdam, The Netherlands; ^4^ Department of Surgical Oncology, Netherlands Cancer Institute, Amsterdam, The Netherlands; ^5^ Department Medical Oncology, Netherlands Cancer Institute, Amsterdam, The Netherlands

**Keywords:** neuroendocrine tumours of the appendix, goblet cell adenocarcinoma, neuroendocrine tumour, appendiceal neoplasms, carcinoid tumour

## Abstract

**Objective:**

Patients with appendiceal neuroendocrine tumours (aNETs) have an excellent prognosis. Appendiceal goblet cell adenocarcinomas (aGCAs), formerly called goblet carcinoid, show overlapping features with aNETs. Many discrepancies exist between studies regarding prognostication of patients with aNETs. In this study, we aim to identify differences in disease course between aNETs and aGCAs to explain inconsistencies in the literature and identify variables influencing recurrence and survival.

**Methods:**

Patients with aNET or aGCA diagnosed between 2000 and 2019 were included. Kaplan–Meier curves were performed in patients with aNET and aGCA independently and in a combined group covering both aNETs and aGCAs to assess progression-free survival (PFS) and disease-specific survival (DSS). Cox regression was used to identify variables influencing PFS and DSS.

**Results:**

In total, 122 patients were included: 92 with aNET and 30 with aGCA. Five- and 10-year PFS rates in patients with aNET were both 98%, whereas in patients with aGCA, this was 57% and 30%, respectively. The 5- and 10-year DSS rates for aNETs were 100 and 96%, and for aGCAs, this was 77 and 58%. In the combined group, 5- and 10-year DSS were 92 and 84%, and the presence of peritoneal metastases showed worse DSS (*P* < 0.001). WHO grade 3 was associated with poorer PFS (HR 18.68, 95% CI (2.24–155.59), *P* = 0.007) and DSS (HR = 10.21, 95% CI (1.23–85.08), *P* = 0.032) in aGCAs.

**Conclusion:**

aNETs and aGCAs are different entities with a distinct prognosis. Differences in DSS between aNETs and aGCAs indicate previous studies misclassified aggressive tumours as aNETs, which addresses the importance of accurate tumour registration and reckoning with changes in nomenclature.

## Introduction

Neuroendocrine neoplasms (NEN) are rare cancers that arise in different parts of the body, with predominant occurrence in the gastroenteropancreatic tract (Dasari *et al.* 2017). In the past decades, the incidence of gastroenteropancreatic neuroendocrine tumours (GEP-NETs) has increased, mainly due to better diagnostic techniques and raised awareness regarding NEN (Das & Dasari 2021).

Appendiceal neuroendocrine tumours (aNETs) are the most common neoplasm of the appendix, with annual incidence rates reported between 0.15 and 1.4 per 100,000 people and a modest female predominance. Most aNETs are small tumours and are discovered incidentally on histopathological examination after appendicectomy for acute appendicitis (In’t Hof *et al.* 2008, Bayhan *et al.* 2020, Twito *et al.* 2021). Patients with aNET generally have an excellent prognosis. However, there is a lot of inconsistency in reported results regarding prognosis, with survival rates ranging from 74 to 100% (Quaedvlieg *et al.* 2001, Hauso *et al.* 2008, Benedix *et al.* 2010, Mullen & Savarese 2011, Brighi *et al.* 2020, Alabraba *et al.* 2021*b*, Twito *et al.* 2021, Storan *et al.* 2023). Especially studies based on national registries, such as the Surveillance, Epidemiology and End Results (SEER) database, report worse survival outcomes for these patients (Sandor & Modlin 1998, McGory *et al.* 2005, Hauso *et al.* 2008, Groth *et al.* 2011, Mullen & Savarese 2011, Madani *et al.* 2017). The incidence of distant metastases has also been a topic of debate. While some studies conclude that aNETs rarely metastasise (Nikhil *et al.* 2018, Holmager *et al.* 2021, Storan *et al.* 2023), others have reported high rates of distant metastases (McGory *et al.* 2005, Madani *et al.* 2017, Landry *et al.* 2021). For instance, Madani *et al.* reported that the incidence of metastatic disease is 3.6%, with peritoneal metastasis (PM) being the most common (69.9%), with an extremely poor 5-year survival rate of only 7% (Madani *et al.* 2017).

Appendiceal goblet cell adenocarcinomas (aGCAs) are tumours that exhibit overlapping features of both NEN and adenocarcinomas of the appendix (Bell & Pai 2022), with annual incidence rates varying from 0.05 to 0.3 per 100,000 people (Palmer *et al.* 2022). Contrary to aNETs, aGCAs behave more aggressively, with 5-year survival rates ranging from 63 to 81% (McCusker *et al.* 2002, Lamarca *et al.* 2016, Alabraba *et al.* 2021*b*, Twito *et al.* 2021, Palmer *et al.* 2022). In addition, up to 18% of patients with aGCA present with distant metastases at diagnosis (Shaib *et al.* 2016, Palmer *et al.* 2022). Up until the 4th WHO classification of tumours of the digestive system, aGCAs were named goblet cell carcinoid and therefore often confused with other types of carcinoid tumours, such as aNETs. The most recent and 5th edition of the World Health Organization (WHO) classification of tumours of the digestive system introduced the term appendiceal goblet cell adenocarcinoma to categorise amphicrine appendiceal neoplasms composed of goblet-like mucinous cells as well as variable numbers of endocrine cells and Paneth-like cells (Assarzadegan & Montgomery 2021). The term goblet cell carcinoid is no longer recommended for these neoplasms.

Observational studies are often based on data from tumour registries, and these studies may include data based on outdated classification systems. Poor outcomes in patients with aNET seem contradictory to clinical experience and we hypothesise that inconsistencies in the current literature are a consequence of obsolete classification of aGCAs as aNETs. In this study, we aim to identify differences in disease progression and prognosis between aNETs and aGCAs, as well as identify variables influencing recurrence and survival. This knowledge will help identify patients most at risk and can contribute to the development of treatment and follow-up guidelines.

## Material and methods

### Study design and subjects

Patients with a histologically proven aNET or aGCA who underwent surgical procedures at baseline, referred to the Netherlands Cancer Institute (NCI) between January 2000 and December 2019, were included. Mixed neuroendocrine non-neuroendocrine neoplasms (MiNEN) were excluded. The NCI is a European Neuroendocrine Tumor Society Centre of Excellence (ENETS centre), and a large portion of all NEN patients in the Netherlands is referred to our hospital. An institutional database has been set up to document and follow up on NEN patients, providing us with the opportunity to perform high-quality studies with a relatively large cohort. Patient demographics and tumour characteristics were retrieved from this longitudinal institutional NEN database. Surgeries were performed either in our tertiary centre or in the referring hospital. In all cases, an expert pathologist of the NCI revised all surgical specimens.

aNETs were classified according to the 5th edition of the WHO classification of tumours of the digestive system, with Ki-67 proliferation index and mitotic count used for grading. NETs of the appendix were categorised as grade 1 (Ki-67% <3% or <2 mitoses per 10 HPF), 2 (Ki-67% between 3 and 20% or 2–20 mitoses per 10 HPF), or 3 (Ki-67% >20% or >20 mitoses per 10 HPF) (Rindi *et al.* 2022). In line with the 5th edition of the WHO classification, aGCAs were classified and graded as adenocarcinomas. The proportion of tubular or clustered growth patterns was used to divide aGCAs into three groups: WHO grade 1, grade 2 or grade 3 (Assarzadegan & Montgomery 2021).

The presence of distant metastases was categorised based on the cancer staging system for appendiceal adenocarcinomas according to the American Joint Committee on Cancer. According to this system, M1b includes PM only, whereas M1c includes distant metastasis to sites other than the peritoneum (Hanna *et al.* 2024). Importantly, patients classified as M1c may also have simultaneous PM. This study was conducted in accordance with institutional ethical guidelines and approved by the Institutional Review Board of the Netherlands Cancer Institute. For this retrospective study, informed consent was obtained from patients where required and waived by the Institutional Review Board where appropriate.

### Objectives

The primary objective of this study was to define differences in progression-free survival (PFS) and disease-specific survival (DSS) between aNETs and aGCAs, considering these as two different disease entities. PFS was defined as the time between histopathological diagnosis from the first surgical procedure and date of recurrence or first progression according to RECIST criteria. DSS was defined as the time between diagnosis and date of death by disease. To study the impact of the most recent WHO classification, aNETs and aGCAs were considered as a single disease entity and analyses were repeated in groups separated based on the presence of PM (M1b). To study the effect of PM only (M1b), patients with M1c were excluded to prevent outcomes driven by distant metastases other than PM. The secondary objective was to identify prognostic factors influencing PFS and DSS for patients with aNET and aGCA.

### Statistics

Statistical analyses were performed using IBM Statistical Package for the Social Sciences (SPSS) software, version 29. Descriptive statistics were used for baseline characteristics. To describe continuous variables (age at diagnosis, tumour size), median with interquartile range (IQR) was used. For categorical variables (sex, performance status (WHO), lymph node involvement, PM (M1b), distant metastases (M1c), grade, and baseline surgery), frequencies and percentages were calculated. Continuous variables were analysed using the independent Mann–Whitney U test. Categorical variables were compared with Chi-square tests. Kaplan–Meier curves were used to analyse PFS and DSS for both the group of patients with aNET and aGCA independently. Analyses were repeated on a combined group of patients with aNET and aGCA, considering them one disease entity. Patients who were still alive at the end of follow-up or who had died of other causes were censored in survival analyses. Log-rank tests were used for comparing survival curves between groups. To identify possible risk factors for recurrence and survival, univariate and multivariate Cox regression were performed. A *P*-value of <0.05 was considered significant.

## Results

### Patients

In total, 122 patients were included in this study, of whom 92 were diagnosed with aNET and 30 with aGCA. Baseline characteristics, as shown in Table 1, were compared between the two groups. The group of patients with aNET differed significantly from the group of patients with aGCA in several baseline characteristics. Patients with aNET were more likely to be younger (43 years (29–57), *P* < 0.001), and less frequently had lymph node metastases (*P* = 0.010), distant metastases (M1c) (*P* < 0.001), and peritoneal metastases (M1b) (*P* < 0.001). One patient with aNET presented with PM (M1b) at baseline. The peritoneal depositions near the diaphragm and hilum of the liver stayed *in situ* after ileocaecal resection. This patient maintained stable disease during a 6-year follow-up. In the group of patients with aGCA, nine patients (30%) presented with PM only, corresponding to M1b disease. In addition, four patients (13.3%) had distant metastases at baseline located other than in the peritoneum (M1c). All four of these M1c patients also had concurrent PM but were categorised as M1c due to the presence of extra-peritoneal sites.

**Table 1:** Baseline characteristics of patients with aNET and aGCA.

Characteristics	aNET	aGCA	*P*-value
	(*n* = 92)	(*n* = 30)	
Sex,* n *(%)			
Male	29 (31.5)	11 (36.7)	0.602
Female	63 (68.5)	19 (63.3)	
Age at diagnosis, median (IQR)	43 (29–57)	56 (48–62)	**<0.001**
Performance status (WHO), *n *(%)			
0	70 (76.1)	20 (66.7)	0.054
1	13 (14.1)	10 (33.3)	
2	1 (1.1)	0 (0)	
Unknown	8 (8.7)	0 (0)	
Lymph node involvement,* n *(%)			
Yes	9 (9.8)	9 (30.0)	**0.010**
No	49 (53.3)	16 (53.3)	
Unknown	34 (37.0)	5 (16.7)	
Distant metastases (M1c),* n *(%)	0 (0)	4 (13.3)	**<0.001**
Including peritoneal metastasis, *n* (%)	0 (0)	4 (13.3)	
Peritoneal metastases only (M1b),* n *(%)	1 (1.1)	9 (30.0)	**<0.001**
Grade NET, *n *(%)			
1	76 (82.6)	N/A	
2	5 (5.4)	N/A	
3	1 (1.1)	N/A	
Unknown	10 (10.9)	N/A	
WHO grade aGCA,* n *(%)			
1	N/A	14 (46.7)	
2	N/A	6 (20.0)	
3	N/A	10 (33.3)	
Tumour size in mm, median (IQR)	11 (8–19)	22 (14–45)	**<0.001**
Baseline surgery,* n *(%)			
Appendectomy only	46 (50.0)	7 (23.3)	**<0.001**
Appendectomy + ileocaecal resection	11 (12.0)	2 (6.7)	
Appendectomy + hemicolectomy	11 (12.0)	1 (3.3)	
HIPEC only	0 (0)	1 (3.3)	
Appendectomy followed by hemicolectomy	18 (19.5)	11 (36.7)	
Appendectomy followed by ileocaecal resection	6 (6.5)	2 (6.7)	
Appendectomy followed by HIPEC	0 (0)	6 (20.0)	

Bold indicates statistical significance. aNET, appendiceal neuroendocrine tumour; aGCA, appendiceal goblet cell adenocarcinoma; IQR, interquartile range

Furthermore, aNETs were likely to have a smaller tumour diameter (11 mm (8–19), *P* < 0.001) compared to aGCAs. The groups did not differ significantly in WHO performance status (*P* = 0.054) and sex (*P* = 0.602), with a female predominance in both groups. Regarding surgical treatment at baseline, patients with aNET were more likely to be treated solely with appendicectomy (50%), and 24% patients underwent a simultaneous ileocaecal resection or hemicolectomy in addition to the appendicectomy. Twenty-four patients (26%) with aNET appeared to have one or more risk factors (e.g. mesoappendiceal invasion or tumour size >2 cm) upon histopathological examination after appendicectomy and were treated with a second operation in agreement with previous European Neuroendocrine Tumor Society (ENETS) guidelines (Pape *et al.* 2016).

In contrast, patients with aGCA more often had additional surgical interventions (63.4%). Seven patients (23.3%) had sole appendicectomy without any additional surgical procedure. Of these patients, three had adjuvant chemotherapy, and in total, nine patients (30%) received adjuvant chemotherapy.

### Survival

Median follow-up was 3.8 years for both aNETs (IQR 0.4–8.6) and aGCAs (IQR 1.5–5.8). Estimation of median PFS and DSS could not be performed in the group patients with aNET due to limited events. One patient with aNET presented with lymph node recurrence 13 years after the initial diagnosis. This patient was treated with sole appendicectomy at baseline followed by an additional hemicolectomy at recurrence and is still alive without disease. Only one patient with aNET died of disease after a follow-up of 7.7 years. This patient was treated with sole hemicolectomy, and histopathological examination showed a tumour of 4 cm and positive lymph nodes. One of the lymph node had extra-capsular growth, was unresectable due to localisation near the superior mesenteric artery, and stayed *in situ*. The tumour was functional with clinical behaviour similar to a serotonin-producing ileum NET, for which the patient was treated with a somatostatin analogue. During follow-up, this patient presented with both locoregional disease progression and distant metastases, including PM.

**Figure 1:**
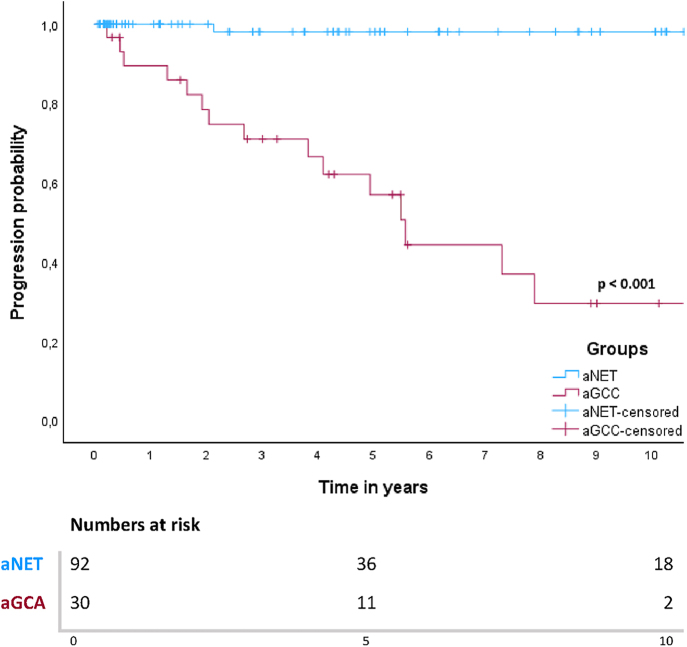
Kaplan–Meier curve for PFS in patients with aNET and aGCA.

For patients with aGCA, median PFS was 5.6 years (95% CI 4.5–6.7) and median DSS was 12.8 years (95% CI 2.3–23.3). Five- and 10-year PFS rates in patients with aNET were both 98%. For aGCA patients, this was 57 and 30%, respectively. Among the twenty patients with aGCA who underwent surgery with curative intent, six patients showed recurrence during follow-up. Ten patients were treated with palliative intent at baseline, and nine of these patients showed progression during follow-up. All patients with disease recurrence or progression had peritoneal tumour depositions either at baseline or at recurrence. Survival curves for PFS for both groups are shown in Fig. 1. A log-rank test defined a significant difference in PFS between patients with aNET and aGCA (*P* < 0.001).

**Figure 2:**
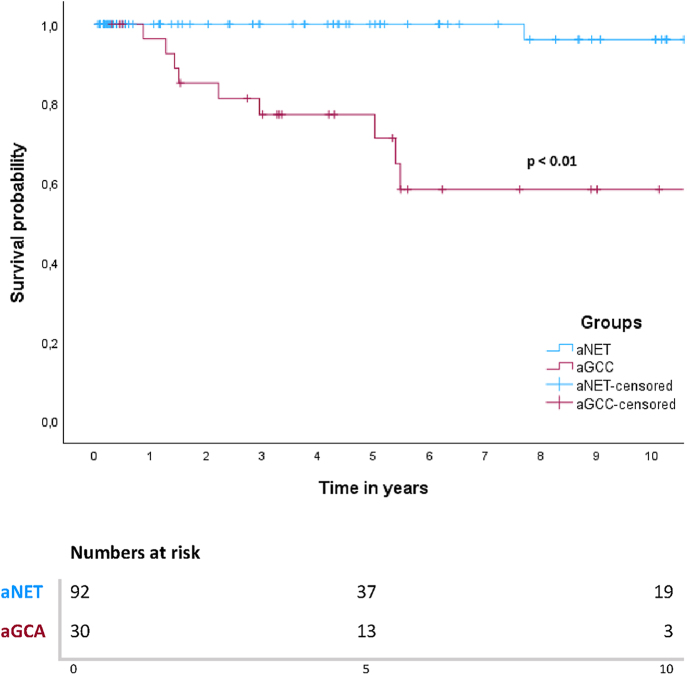
Kaplan–Meier curve for DSS in patients with aNET and aGCA.

**Figure 3:**
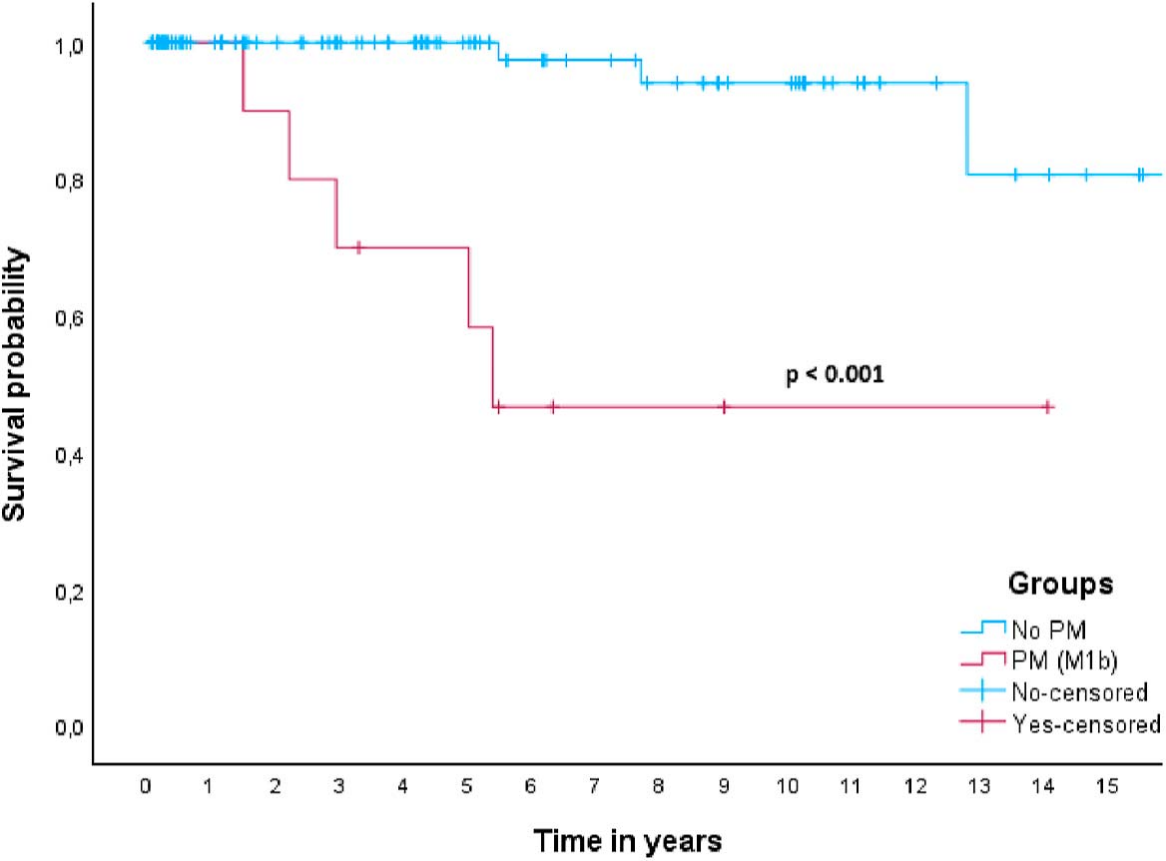
Kaplan–Meier curve for DSS in patients with and without peritoneal metastasis (M1b).

In this cohort, 5- and 10-year DSS rates for patients with aNET were 100 and 96%. The 5- and 10-year DSS rates for patients with aGCA were 77 and 58%. In the aNETs group, only one patient died of disease, as mentioned above. In the aGCAs group, ten patients (33.3%) died of disease after progression during follow-up, all of whom had peritoneal metastases either at baseline or during follow-up. One out of 14 patients (7%) with aGCA WHO grade 1 died of disease, whereas 50% patients with WHO grade 2 and 33% with WHO grade 3 died of disease. Survival curves showed a significant difference in survival between patients with aNET and aGCA (*P* < 0.001) (Fig. 2). When all patients were grouped together (*n* = 122), median 5- and 10-year DSS survival were 92 and 84%. When patients were subdivided based on the presence of PM only (M1b) at baseline or absence of distant metastases (M0), there was a significant difference in survival based on a log-rank test (*P* < 0.001) (Fig. 3). Patients with M1c disease (*n* = 4) were excluded from this analysis, leaving 118 patients. Of these patients, 108 did not have PM at baseline, and only two (1.9%) of these patients died of disease. Five out of nine patients (55.6%) with PM (M1b) died of disease, with a median DSS of 5.4 years and a 5-year DSS rate of 64%.

### Prognostic factors for PFS and DSS in patients with aGCA

**Figure 4:**
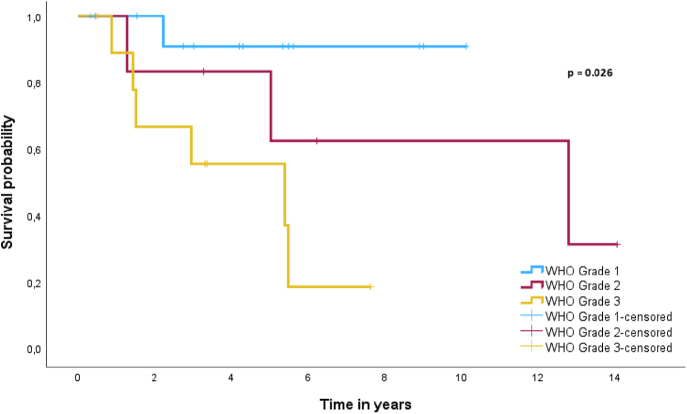
Kaplan–Meier curve for DSS in patients with aGCA subdivided based on WHO grade.

Due to the limited number of patients with disease progression or death in the aNETs group, prognostic factors for PFS and DSS could not be established. Factors with predictive potential for PFS and DSS for patients with aGCA are shown in Table 2. Univariate Cox regression revealed WHO grade 3 as a significant predictor for poorer prognosis for both PFS (HR = 18.68, 95% CI (2.24–155.59), *P* = 0.007) and DSS (HR = 10.21, 95% CI (1.23–85.08), *P* = 0.032). However, WHO grade 3 had no significant prognostic implications for both PFS (HR = 3.52, 95% CI (0.95–13.28), *P* = 0.062) and DSS (HR = 2.71, 95% CI (0.54–13.64), *P* = 0.226) when compared with WHO grade 2. To demonstrate the effect of WHO grade on DSS in patients with aGCA, a Kaplan Meier curve was used as shown in Fig. 4. A log-rank test demonstrated a significant difference in survival between WHO grades based on the newest classification of aGCA (*P* = 0.026). Multivariate Cox regression was not performed due to the limited number of events.

**Table 2:** Univariable Cox regression for PFS and DSS in patients with aGCA.

Characteristics	PFS				DSS		
	*n*	HR	95% CI	*P*-value	HR	95% CI	*P*-value
Sex							
Male	11	1			1		
Female	9	1.19	(0.36–3.93)	0.776	1.47	(0.37–5.78)	0.581
Age at diagnosis	30	1.003	(0.95–1.06)	0.925	1.01	(0.94–1.08)	0.856
Lymph node involvement							
No	16	1			1		
Yes	9	1.12	(0.96–9.77)	0.144	3.86	(0.96–15.60)	0.097
Unknown	5	0.71	(0.47–8.64)		1.11	(0.11–10.93)	
M status							
M0	17	1			1		
M1b	9	2.72	(0.82–9.00)	0.102	4.21	(0.81–22.00)	0.087
WHO grade							
1	14	1			1		
2	6	7.28	(0.81–65.50)	0.076	3.81	(0.35–42.12)	0.275
3	10	18.68	(2.24–155.59)	**0.007**	10.21	(1.23–85.08)	**0.032**

Bold indicates statistical significance. aGCA, appendiceal goblet cell adenocarcinoma; PFS, progression-free survival; DSS, disease-specific survival.

## Discussion

This large cohort study in two rare cancers confirms that patients with aNET have a significantly better prognosis as compared to those with an aGCA, when taking into account a proper segregation of both neoplasms. The excellent prognosis of patients with aNET observed in this study aligns with previous institutional studies (Brighi *et al.* 2020, Alabraba *et al.* 2021*b*, Twito *et al.* 2021, Nesti *et al.* 2023, Storan *et al.* 2023). Although some studies reported worse survival outcomes for patients with aNET (Sandor & Modlin 1998, McGory *et al.* 2005, Hauso *et al.* 2008, Groth *et al.* 2011). These studies were based primarily on national registries such as the Netherlands Cancer Registry (NKR) or the SEER database. In our study, presence of PM (M1b) was associated with worse survival outcomes compared to patients without metastatic disease (M0), with 5-year survival rates dropping to 64%. Baseline characteristics showed that PM were predominantly present in patients with aGCA, indicating that these tumours are a different entity and reinforcing the newest WHO guidelines for excluding aGCAs from the group of NETs (Assarzadegan & Montgomery 2021). WHO grade 3 seems to be a predictive factor for progression and poorer survival in patients with aGCA.

Patients with aNET were less likely to present with lymph node metastasis at baseline compared to patients with aGCA (*P* = 0.010). However, information on presence of lymph node involvement was missing in a substantial portion of the cohort; 34 patients with aNET (37.0%) and five patients with aGCA (16.7%). This lack of information is mainly attributable to treatment modality, as lymph node assessment is often not performed with appendicectomy only. In our study, 34 patients with aNET and four patients with aGCA without information on lymph node involvement were treated with sole appendicectomy. To mitigate this problem, Nesti *et al.* constructed a logistic regression model to estimate the probability of residual lymph node metastases after appendicectomy in patients with aNETs measuring 1–2 cm (Kaltsas *et al.* 2023, Nesti *et al.* 2023). They estimated that approximately 13% of these patients might have had lymph node involvement. Importantly, they also reported that regional lymph node metastases for aNETs between 1 and 2 cm are not associated with poorer survival and have limited clinical relevance.

Prognostication of patients with aNET varies significantly between different studies depending on the definition of aNET (Quaedvlieg *et al.* 2001, Hauso *et al.* 2008, Benedix *et al.* 2010, Mullen & Savarese 2011, Brighi *et al.* 2020, Alabraba *et al.* 2021*b*, Twito *et al.* 2021, Storan *et al.* 2023). In our study, when aNETs and aGCAs were considered a single disease entity, survival rates approximate worse outcomes as reported in the previous literature (Sandor & Modlin 1998, McGory *et al.* 2005, Hauso *et al.* 2008, Groth *et al.* 2011, Mullen & Savarese 2011). This supports our hypothesis that especially prior population-based studies likely included aggressive tumours, leading to skewed results regarding the epidemiology of aNETs. Changes in classification and nomenclature of tumours of the appendix could have contributed to misclassification and inclusion of more aggressive tumours in studies, as aGCAs were formerly named goblet cell carcinoid of the appendix and often grouped with NETs.

A poorer outcome of patients with aGCA is supported by the previous literature, with 5-year survival rates varying between 63 and 81% (Turaga *et al.* 2012, Lamarca *et al.* 2016). The extended variability regarding survival outcomes may be attributable to distribution of patients among different medical centres. In a tertiary referral hospital, a pre-selection of patients more at risk for recurrence or patients eligible for extensive treatment such as hyperthermic peritoneal chemotherapy (HIPEC) could influence the formation of such a cohort. Despite our study being performed in a tertiary centre, our results show survival outcomes approximating average rates reported in the current literature, with a proportionate distribution of tumours with favourable and less favourable characteristics. An important methodological difference in our study is the use of DSS instead of overall survival (OS), which is a more sophisticated way to demonstrate cancer survival outcomes. Especially in a rare disease with small cohorts, using OS can possibly lead to bias regarding prognostic results. In our cohort, only one patient died of disease in the group of patients with aNET, while ten other patients died of a cause different from the neuroendocrine tumour. In the group with aGCA, all patients who died during follow-up died due to their disease.

Multiple previous studies reported high rates of distant metastases in patients with aNETs (McGory *et al.* 2005, Landry *et al.* 2021). Our study, on the contrary, shows that aNETs rarely metastasises, which is in conclusion with other institutional studies (Madani *et al.* 2017, Nikhil *et al.* 2018, Holmager *et al.* 2021). In the study by Madani *et al.* patients with aNETs and PM had the worst survival outcomes of all patients with GEP-NETs, with 5-year survival rates of only 7% (Madani *et al.* 2017). In our study, survival analysis in patients with PM (M1b), when aNETs and aGCAs were considered a single disease entity, showed a 5-year DSS rate of 64%. Baseline characteristics of our study, however, indicate that PM were predominantly present in patients with aGCA. Accompanied by clinical experience that aNETs almost never metastasise, this suggests that in the study of Madani *et al.* patients with aGCA have been included in the survival analyses for aNETs and have influenced survival outcomes based on misclassification bias. The large discrepancy regarding survival rates for patients with PM (M1b) between our study and the study of Madani *et al.* is likely due to the inclusion of other distant metastases (M1c). Their study showed a significant contribution of M1c disease to worse survival, but still included patients with M1c in their survival analyses regarding PM only (M1b). Contradictions in the literature regarding NETs of the appendix are often attributed to issues regarding classification and registration. This again emphasises the importance of proper segregation and registration of tumours to retain trustworthy outcomes.

In our cohort, establishing prognostic factors for aNETs was not feasible due to the limited number of events. In patients with aGCA, WHO grade 3 showed to be a predictor for both poorer survival and progression. The last decades there have been multiple different classification systems for aGCAs. All of these systems have been able to stratify patients into prognostic groups based on proposed grading (Tang 2008, Lee *et al.* 2015, Lamarca *et al.* 2016, Nonaka *et al.* 2018). To our knowledge, we are the first to show the prognostic value of the newest WHO classification in a complete cohort of patients with aGCA. In our study, baseline characteristics show that presence of lymph node involvement at baseline was less common than presence of distant metastases, which is in conclusion with the previous literature (Tang 2008). This suggests that metastatic disease in aGCA is not exclusively driven by lymphogenous spread, as aGCAs preferably metastasise to the peritoneum and the ovaries in female patients (Tang 2008, Lamarca *et al.* 2016, Tsang *et al.* 2018). Higher graded aGCAs are associated with an increased risk of PM and therefore poorer survival outcomes (Taggart *et al.* 2015, Shyu *et al.* 2020). In the present cohort, all patients with disease recurrence and all patients who died of their disease had PM either at baseline or during follow-up. Presence of PM (M1b) was associated with worse DSS as compared to patients without metastatic disease (*P* < 0.001). In a Cox regression, however, PM could not be appointed as a predictor for worse survival in the total cohort of patients with aGCA, probably due to the limited number of patients and events in this study.

The ENETS and NANETS both support right-sided hemicolectomy in patients with aGCA (Boudreaux *et al.* 2010, Pape *et al.* 2012). However, the high rates of recurrence and poor prognosis justify a more aggressive approach. A study by Alabraba *et al.* reported a very poor prognosis for patients with aGCA despite completion surgery (Alabraba *et al.* 2021*a*), questioning current treatment recommendations. The large number of patients in our study with PM at recurrence suggests treatment with HIPEC should be considered for patients with aGCA after completion surgery. Because peritoneal tumour depositions are correlated with increasing grade, the question arises whether there should be distinct treatment guidelines for patients with WHO grade 1 aGCA versus patients with a higher WHO grade, proposing omission of right-sided hemicolectomy for patients with grade 1 and post-operative HIPEC for grade 2 or 3 (Kowalsky *et al.* 2021). Determination of prognostic factors for patients most at risk is therefore even more important. However, small cohorts, inconsistencies in nomenclature and changing grading systems for aGCA have made this a challenge.

To understand the interpretation of the results of this study, some limitations need to be addressed. First, this study comprises a retrospective cohort, which is subject to precise and accurate record keeping. However, in our study, pathology reports were complete as all patients’ surgical specimens were revised by a specialised pathologist based on the latest WHO guidelines. Second, even though our hospital is a tertiary referral centre for patients with aNET and aGCA, the present cohort had limited numbers of patients, reflected by the wide confidence intervals and limited number of events. Despite the small cohort for aGCAs, it is still a relatively large group for this rare disease, including between 3 and 20% patients in the Dutch population, exceeding case study reports. Third, the relatively short follow-up period for both aNET and aGCA may have resulted in an underestimation of events. In the group of patients with aNET, a short follow-up is justified based on current guidelines. Patients treated with sole appendicectomy without further risk factors do not require active surveillance (Kaltsas *et al.* 2023). The low number of events in this group reflects the indolent course of disease and aligns with the previously published literature, making it unlikely that clinically relevant events were missed. The median follow-up length for patients with aGCA (3.8 years) did not extend beyond median PFS (5.6 years) and DSS (12.8 years), which could have led to underestimation of long-term events. Most aGCA-related deaths occurred within the first 2 years after diagnosis and were therefore captured within the follow-up period. Within our centre, patients without evidence of disease were followed for 5 years after diagnosis. A small subset of patients received extended follow-up due to risk factors such as PM or lymph node involvement. Only a limited number of patients were still at risk at median PFS and DSS, and risk estimation beyond 5 years should be interpreted with caution. Finally, despite a relatively large cohort of aNETs, the small number of events prevented us from determining prognostic factors and prevented evaluation of patients most at risk. This is mainly due to the excellent prognosis of patients with aNET, and even larger cohorts would experience difficulties analysing this data.

## Conclusion

This study contributes to the understanding that aNETs and aGCAs are two different disease entities with a completely distinct prognosis, underscoring the 5th WHO classification for excluding aGCAs from the group of NEN. It also highlights inconsistencies in the current literature, particularly in national registry-driven studies. We demonstrate inclusion of more aggressive tumours in previous epidemiological research on aNETs. It is extremely important to reckon with definitions and nomenclature before translating the conclusions of these studies to the intended group of patients. Studies regarding rare cancers and small cohorts are especially prone to large differences in outcome when registration of tumours is inaccurate. Future retrospective studies regarding aGCAs should preferably consist of large multicentre cohorts to confirm that PM and WHO grade indeed predict worse survival in patients with aGCA, and whether current treatment guidelines should be adjusted to preclude peritoneal tumour depositions at recurrence.

## Declaration of interest

There is no conflict of interest that could be perceived as prejudicing the impartiality of the research reported.

## Funding

This work did not receive any specific grant from any funding agency.

## Author contribution statement

KD made a substantial contribution on collection of data, designing the study, analysis, interpretation of the study and writing the manuscript. JB made a substantial contribution on revision of pathology samples and reviewing the results and manuscript. KK made a substantial contribution on reviewing the results and manuscript. GD made a substantial contribution designing the study, reviewing data analysis, interpretation of results and reviewing the manuscript. MT made a substantial contribution designing the study, reviewing data analysis, interpretation of results and reviewing the manuscript.

## Ethics

This study was conducted in accordance with institutional ethical guidelines and approved by the Institutional Review Board of the Netherlands Cancer Institute. For this retrospective study, informed consent was obtained from patients where required, and waived by the Institutional Review Board where appropriate.
